# Role of Polo-like Kinases Plk1 and Plk4 in the Initiation of Centriole Duplication—Impact on Cancer

**DOI:** 10.3390/cells11050786

**Published:** 2022-02-24

**Authors:** Ingrid Hoffmann

**Affiliations:** F045, Cell Cycle Control and Carcinogenesis, Im Neuenheimer Feld 242, 69115 Heidelberg, Germany; ingrid.hoffmann@dkfz.de

**Keywords:** centrosome, centriole duplication, Plk4, centriole disengagement

## Abstract

Centrosomes nucleate and anchor microtubules and therefore play major roles in spindle formation and chromosome segregation during mitosis. Duplication of the centrosome occurs, similar to DNA, only once during the cell cycle. Aberration of the centrosome number is common in human tumors. At the core of centriole duplication is the conserved polo-like kinase 4, Plk4, and two structural proteins, STIL and Sas-6. In this review, I summarize and discuss developments in our understanding of the first steps of centriole duplication and their regulation.

## 1. Introduction

The centrosome is a membrane-less organelle and functions as the main microtubule-organizing center in animal cells for mitotic spindle assembly and cilia/flagella formation [1,2]. During interphase, centrosomes nucleate the formation of the microtubule cytoskeleton, and during mitosis, they form the spindle poles of the bipolar mitotic spindle [3,4].

A mature centrosome typically comprises a pair of centrioles embedded in a protein matrix, the pericentriolar material (PCM). Centrioles are cylindrical structures that are made up of nine groups of three microtubules, so-called triplet microtubules, which are linked together to mark the walls of the cylinder. PCM contains a large number of proteins, such as the γ-tubulin ring complex (γ-TuRC), Cdk5Rap2, Cep192 and pericentrin (PCNT) [5]. Similar to DNA replication, centrosome duplication normally occurs once per cell cycle and is initiated in G1-S phase by assembling a single daughter centriole (procentriole) near the wall of the mother centriole in a strictly regulated process. Proper control of centrosome duplication ensures accurate chromosome separation during cell division and ciliogenesis. The dysregulation of centriole duplication has been observed in conditions as microcephaly and cancer [6]. In recent years, much progress has been made in our understanding of how the centrosome is assembled and duplicated, and how these processes are deregulated in human disease. Evolutionarily conserved core factors of centriole duplication that have been described so far include Plk4, STIL/Ana2 and Sas-6 [1,7,8,9]. After the new procentriole has formed, PCM is recruited to this centriole in a step called centriole-to-centrosome conversion.

## 2. “Licencing” Centrioles for a New Round of Centriole Duplication

Similar to DNA replication, which depends on the origin licencing machinery, centrioles are only able to duplicate when they have passed through mitosis [10]. The two centrioles are engaged during mitosis but need to be separated (centriole disengagement) during exit from mitosis, and this disengagement is required for centriole duplication in the next cell cycle. Early electron microscopy studies show that upon entry into G1, the engaged centriole pairs lose their tight orthogonal configuration and disengage, which ‘licences’ the centrioles for the next round of centrosome duplication [11]. Centriole disengagement allowing the re-duplication of the parental (mother) centriole is the first step that is required for splitting the pair of centrioles and subsequent duplications of the centriole. Cdk5Rap2 (also known as Cep215), a member of the CNN family of proteins, is required to maintain centriole engagement and cohesion, thereby restricting centriole duplication. This has been demonstrated in loss-of-function Cdk5Rap2 mutant MEFs, as centrioles were disengaged and lost the normal paired configuration [12]. Interestingly, the same machinery that regulates sister chromatid separation also regulates centriole disengagement and licencing. Centriole disengagement is mediated by separase and polo-like kinase 1 (Plk1) and occurs downstream of checkpoint silencing and APC/C activation [13,14]. Centrioles in separase -/- cells do not disengage at the correct point of the cell cycle, but eventually disengage as a result of Plk1 activity (Tsou et al., 2009). Further experiments are required to clarify the role of cohesin in centriole engagement as cohesin cleavage is not sufficient for centriole disengagement in *Drosophila* embryos [15]. A likely substrate for separase in centriole disengagement is pericentrin/kendrin (PCNT) [16,17]. PCNT is cleaved by activated separase at a consensus site in vivo and in vitro, and this leads to the delayed release of PCNT from the centrosome later in mitosis. Furthermore, the expression of a non-cleavable PCNT mutant suppresses centriole disengagement and subsequent centriole duplication [17]. Degradation of the centrosomal linker protein Cep68 leads to the removal of Cdk5Rap2, which is localized at the peripheral PCM to prevent centriole separation prior to disengagement [18]. It seems that both Cdk5Rap2 and PCNT are both required for centriole engagement, but how exactly they collaborate still needs to be clarified.

Studies using correlative live/electron microscopy demonstrated that Plk1 induces maturation and distancing of the daughter centriole. This allows reduplication of the mother centriole, even if the original daughter centriole is still orthogonal to it [19]. It therefore is likely that centriole distancing occurs due to gradual Plk1-dependent maturation of the daughter centriole, leading to accumulation of PCM components around its proximal parts. This in turn stimulates distancing from the mother centriole. During mitosis, centriole engagement is dependent on Cep57, as the depletion of Cep57 causes precocious centriole disengagement. Interestingly, Cep57 has been identified as a binding partner of PCNT. Their interaction is required for the proper localization pattern of PCNT to organize mitotic PCM [20]. Thus, the maintenance of centriole engagement during mitosis is dependent on the Cep57–PCNT interaction. Recently, it has also been shown that a paralogue of Cep57, Cep57l1, together with Cep57 redundantly regulates centriole engagement during interphase, as the co-depletion of both proteins causes centriole disengagement during interphase and centriole-reduplication [21]. Precocious centriole disengagement in interphase leads to a release of mother centrioles from a block of reduplication [22]. Accordingly, the double depletion of Cep57 and Cep57l1 promotes a higher frequency of multipolar spindle formation and chromosome instability than the single depletion of Cep57 [20,21].

**Figure 1:**
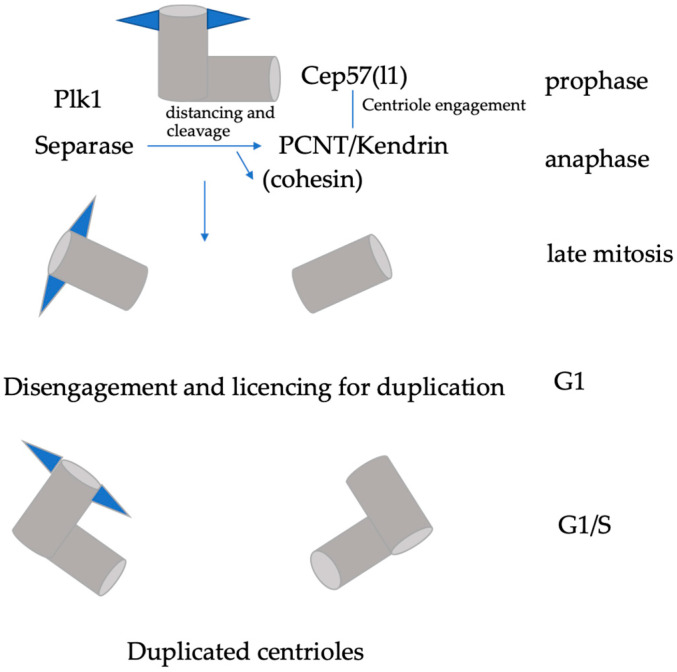
Regulation of centriole disengagement. Maintenance of centriole engagement in mitosis is dependent on the interaction between Cep57 and PCNT/kendrin, and in part by cohesin. Plk1 activity promotes distancing of centrioles. Cleavage of PCNT by separase induces centriole disengagement and licencing for centriole duplication.

Thus, the centrosome duplication cycle depends on the timely activation of the anaphase-promoting complex (APC/C) and separase activity. The APC/C is an E3 ubiquitin ligase, and its major function is to trigger the metaphase-to-anaphase transition, upon proper attachments of kinetochores to the mitotic spindle. APC/C binds to Cdc20 and targets securing for degradation. This step liberates the protease separase, which in turn destroys cohesion between sister chromatids. Mitotic delay caused by the spindle-assembly checkpoint (SAC) triggers the initiation of centriole duplication licencing in response to precocious centriole disengagement [23], followed by centrosome fragmentation. Upon the appearance of active Plk1 (pT210) at the centrosome, procentrioles mature, disengage from mother centrioles, and ultimately duplicate, but Plk1 itself is not responsible for procentriole assembly [22]. Proper control of centriole disengagement is necessary to prevent the premature onset of centriole duplication (Figure 1).

## 3. Recruitment of Plk4 by Cep152 and Cep192 to the Centrosome

**Figure 2:**
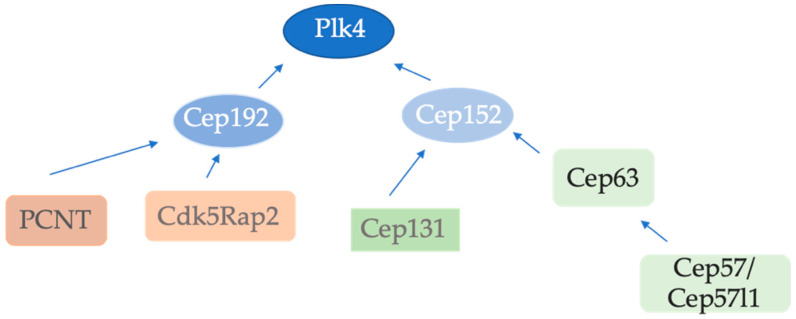
Recruitment of centriole duplication proteins to the centrosome. Plk4 is recruited by both Cep152 and Cep192. PCNT and Cdk5Rap2 recruit Cep152 whereas Cep152 recruitment to the centrosome is mediated by Cep131 and the Cep63/Cep57(Cep57l1) complex. Cep192 is recruited to mitotic centrosomes by PCNT and Cdk5Rap2.

To initiate centriole duplication in human cells, Plk4 must be recruited to the centrosome. This step occurs in a hierarchical order by two distinct scaffolds, Cep192 and Cep152 [24,25,26]. Cep192 and Cep152 are both PCM proteins but show distinct localization. Cep152 localization is confined to the proximal half of a centriole while Cep192 localizes along the entire wall of both mother and daughter centrioles [24,25,27]. Plk4 consists of an N-terminal catalytic kinase domain and a C-terminal domain containing three polo-boxes (PB) which regulate Plk4 localization and function [28]. Cep152 and Cep192 interact with polo-boxes PB 1+2 (cryptic polo-box) of Plk4 in a competitive and mutually exclusive way [27]. Cep152 depletion alone does not significantly decrease Plk4 levels at the centrosome, whereas the depletion of both Cep192 and Cep152 enhances the Plk4 localization defect [25], suggesting that Cep152 and Cep192 co-operate to recruit Plk4 to the centrosome in order to trigger centriole duplication. In another study, it was demonstrated that the disruption of either the Cep192–Plk4 interaction or the Cep152–Plk4 interaction impedes centriole duplication [26]. Cep192 is supposed to bind to Plk4 first. Then, Plk4 is transferred to the N-terminus of the Cep152 scaffold where it remains localized [26]. It is currently unclear how this first step of Plk4 binding by Cep192 is regulated. Loss of either Cep152- or Cep192-dependent interactions with Plk4 impairs the recruitment of Sas-6, a coiled-coil protein required for cartwheel formation [26]. The exact role of Cep192 in centriole duplication, however, is not clear, as centrioles can still initiate in the absence of Cep192 [25,26,29]. However, Cep192 seems to be critical for centriole-to-centrosome conversion and is also important for organizing the PCM around the growing centriole [30]. Together, in mammalian cells, Cep152 and Cep192 appear to function as distinct and independent scaffolds to localize Plk4 and promote its critical role in the initiation of centriole duplication (Figure 2).

It seems that in other organisms, only one scaffold is required for Plk4 recruitment to the centrosome. Studies in *D. melanogaster* have demonstrated that the centriolar protein Asterless (Asl; ortholog of mammalian Cep152) provides a conserved molecular platform, the amino terminus of which interacts with polo-boxes 1 + 2 of Plk4 [31]. Work in *C. elegans* has revealed that the recruitment of Zyg-1 (the Plk4 ortholog) is mediated by the coiled-coil protein Spd-2 (ortholog of the mammalian Cep192) in the mother centriole [7,32,33]. A list of centrosomal proteins named in this review and their orthologs is found in Table 1.

## 4. Regulators of Cep192 and Cep152 Recruitment to the Centrosome

The centriole and the PCM scaffold proteins, PCNT and Cdk5Rap2, co-operate to recruit Cep192 to the spindle poles in mitosis in order to facilitate bipolar spindle formation [29,50], and is present at both interphase and mitotic centrosomes [29]. Depletion of Cep192 leads to a strong but not complete loss of centrosomal Cep152, while depletion of Cep152 does not influence Cep192 localization [25,26]. It is unclear how exactly Cep192 reduces centrosomal Cep152 levels, and whether the interaction between the two proteins is direct [26]. For centrosomal loading, it was also shown that Cep152 requires Cep63 and Cep57 (both Cep57 and its paralog Cep57l1). Cep63 is required for maintaining normal centrosome numbers [51,52,53]. Cep63 and Cep152 interact and co-localize at a discrete ring around the proximal end of the parental centrioles [51,52,53]. Cep57 and Cep57L1 colocalize with Cep63 and Cep152 and form a stable complex at the proximal end of the parental centrioles in both cycling and multicilitated cells undergoing centriole amplification. The depletion of both proteins, but not either one alone, blocks loading of Cep63 to centrioles [54]. Cep57/Translocin is evolutionarily conserved from human to *Trypansoma cruzi* [20], but it is not clear whether the proteins from different organisms function in the same fashion. Cep57 has been described as an FGF-2 binding and trafficking protein required for proper chromosome segregation [55], where it regulates the loading of the Mad1–Mad2 complex at kinetochores [56]. It has also been reported that Cep57 is responsible for mosaic variegated aneuploidy (MVA) [57]. It seems that Cep57 is localized to kinetochores and/or centrosomes. The existing data for centrosome localization suggest that Cep57 is a PCM component and that its depletion leads to multipolar spindle formation which might be caused by PCM fragmentation [58]. Recent data reveal that the overexpression of Cep57 rapidly stimulates centriole overduplication and mitotic defects [59]. Selective chemical crosslinking experiments demonstrate that Cep57, Cep63, and Cep152 are parts of a ring-like complex which localizes around the procentriole close to the cartwheel [60]. The nine-fold symmetrical cartwheel is a subcentriolar structure consisting of a central hub and nine radially arranged spokes which are located at the proximal end of the centriole. It appears in the initial stage of centriole assembly as the first nine-fold symmetrical structure. Cep57 binds to the well-conserved PACT-domain of PCNT and forms ring-like structures around the mother centriole wall [20]. Cep57 directly interacts with Cep63 but is dispensable for centriolar anchoring of the Cep63–Cep152 complex [54]. Moreover, Cep57l1, the paralog of Cep57, which is conserved in vertebrates, forms a complex with Cep63–Cep152 at the proximal end of centrioles. Cep57l1 has an ~43% sequence identity to Cep57 and similar function domains. Cep57 and Cep57l1 appear to act redundantly to recruit Cep63–Cep152 for centriole duplication (Figure 2). Only their co-depletion, but not either one alone, leads to a block in the loading of Cep63–Cep152 to the mother centriole, and impairs the onset of centriole duplication as hSas-6 loading is impaired [54].

Asterless (Asl), the D. melanogaster ortholog of Cep152, is loaded during centriole-to-centrosome conversion by Ana1 [61]. The initial incorporation of Asl into newly formed centrioles depends on D-Sas4 [62]. So far, no data are available from human cells on Cep152 loading by their human orthologs, Cep295 and CPAP.

The centriolar satellite component Cep131 has also been demonstrated to recruit Cep152 to the centrosome. Cep131 interacts in vivo with Cep152. Moreover, the depletion of Cep131 greatly reduces Cep152 levels at the centrosome [63]. Disrupted centrosomal localization of Cep152 was also observed in MEFs from Cep131^gt/gt^ embryos [51]. So far, no role of Cep152 in centriolar satellites has been demonstrated. Similar to Cep152, Cep131 siRNA treatment of cells or Cep131 KO MEFs lead to centriole duplication defects.

It is still unclear how the depletion of Cep192 reduces Cep152 levels at the centrosome, as centrosomal loading of Cep152 depends on Cep63 and Cep57/Cep57l1. Therefore, the detailed and temporal recruiting steps of Cep152 to the centrosome still need to be clarified.

## 5. Formation of a New Centriole

Once recruited to the centrioles, the levels and activity of Plk4 need to be tightly regulated. Plk4 forms a stable homodimer. PB 1 and 2 domains in Plk4 are required for dimerization and centriole recruitment [24,25,26,31,35,64]. Trans-autophosphorylation within the dimer causes low steady-state levels and triggers the E3 ligase SCF-β-TrCP-mediated protein degradation of Plk4 [65,66,67,68,69]. Degradation of Plk4, mediated by SCF-β-TrCP, is triggered by the phosphorylation of the DSG motif, a destruction motif located within the linker 1 (L1) region of Plk4 [66,68,70,71]. In the early G1 phase of the cell cycle, Plk4 first appears as a ring-like pattern surrounding the centrioles [26,72]. The binding of STIL to Plk4 induces Plk4 transition from the ring-like pattern to a single dot at the G1/S phase. Since STIL binds to the linker region in the N-terminus of Plk4, which contains the recognition motif of for SCF-β-TrCP-mediated degradation, it is conceivable that STIL binding to this part of Plk4 may preclude recognition by and interaction with β-TrCP, leading to Plk4 stabilization [73]. The Plk4–STIL interaction leads to a conformational change in Plk4 and subsequent kinase activation [73,74,75]. Activation of Plk4 occurs on a threonine residue within the activation domain (T-loop) [75,76,77,78]. STIL is then phosphorylated by activated Plk4 within its C-terminal STAN motif. Subsequently, a key structural component, Sas-6, is recruited, which initiates the assembly of a cartwheel [72,74,79,80]. The protein Sas-6 is found at the hub of the cartwheel and forms the structural basis for the procentriole [81,82]. STIL also interacts with CPAP, a protein important for centriole elongation [37,83,84,85]. Centriole length must be carefully regulated to restrict procentriole numbers and thus ensure accurate cell division [86].

## 6. Centrosome Defects and Cancer

A broad range of human cancers exhibit centrosome abnormalities. They are correlated with advanced tumor grades and poor prognosis [87,88,89]. Centrosome abnormalities are either structural or numerical and can co-exist in tumors. Structural aberrations comprise alternations in the size and shape of centrosomes [90]. For example, severe centriole over-elongation can promote amplification through both centriole fragmentation and ectopic procentriole formation [86], but can also cause metastasis [90]. Centrosome amplification can lead to chromosomal instability through increasing the rates of chromosome mis-segregation and the formation of micronuclei [91,92,93]. Extra centrosomes cause the formation of multipolar spindles, as each of them is capable of nucleating microtubules. Multipolar divisions lead to high levels of chromosome mis-segregation and cell death [91]. Tumor cells may suppress multipolar divisions by clustering supernumerary centrosomes to form a pseudo-bipolar mitotic spindle [94]. Chromosome segregation errors are induced by incorrect merotelic attachments between kinetochores and microtubules [91,95]. Amplified centrosomes can also change the interphase microtubule cytoskeleton, leading to an increase in cell invasion and metastasis [88,89].

The first trailblazing work on the impact of Plk4 overexpression in cancer came from studies in Drosophila, showing that centrosome amplification cannot promote spontaneous tumors. However, neuroblast and epithelial cells with supernumerary centrosomes can initiate tumorigenesis when they are transplanted into wild-type flies [96]. In human cells, the situation is more complex, as initial studies did not observe the formation of spontaneous tumors in mice upon high overexpression of Plk4 [97,98,99]. A modest increase in Plk4 protein levels leading to higher centrosome numbers induced the formation of spontaneous tumors [100]. Spontaneous lymphomas that develop in response to the presence of extra centrosomes show the downregulation of p53 genes. Therefore, tumors that develop spontaneously upon centrosome amplification show an impairment of the p53 pathway [100].

What remains to be analyzed in the future are the key mechanisms by which centrosome defects contribute to tumor formation and/or progression. Centrosome defects, both numerical and structural, can promote distinct changes in cell physiology and behavior. While numerical centrosome aberrations have been well studied, it will be interesting to find out how structural centrosomal aberrations trigger tumorigenesis and metastasis. For example, how overly long centrioles lead to increased microtubule nucleation activity and stability, and why numerical and structural abnormalities often coincide in tumors.

## Figures and Tables

**Table 1:** Mammalian proteins and their orthologs involved in the early steps of centriole duplication.

Mammals	*D. melanogaster*	*C. elegans*	References
Plk4	Sak	Zyg-1	[34]
			[1]
			[7]
Cep152	Asterless		[24]
			[35]
			[31]
Cep192	Spd-2	Spd-2	[33]
			[32]
			[36]
STIL	Ana-2	Sas-5	[37]
			[8]
			[38]
			[9]
Cdk5Rap2	CNN	Spd-5	[39]
			[40]
Sas-6	Sas-6	Sas-6	[41]
			[42]
			[43]
CPAP	Sas-4	Sas-4	[44]
			[45]
			[46]
Cep295	Ana-1		[47]
			[48]
			[49]
